# Polarised upgrading: the changing occupational structure of large cities in Germany and the UK, 1991–2021

**DOI:** 10.1186/s12651-025-00420-2

**Published:** 2025-12-20

**Authors:** Daniel Oesch, Katy Morris, Gina-Julia Westenberger

**Affiliations:** 1 LIVES Centre, University of Lausanne, Lausanne, 1015 Switzerland; 2 SOFI, Stockholm University, Stockholm, 10691 Sweden

**Keywords:** Global cities, Urban labour markets, Occupational change, Class structure, Employment, J21, J40, P25

## Abstract

An influential thesis predicted in the 1990s that large cities would become polarised as both high-wage professional and low-wage service jobs expanded at the expense of middle-wage workers. We revisit this thesis by analysing change in the occupational class structure of the ten largest cities in Germany and the UK, 1991–2021. Using UK census and German social security data (SIAB), we find for all cities strong job growth at the top – among professionals and managers –, moderate growth at the bottom – among service and sales workers – and a sharp decline in the middle – among office clerks and production workers. The result is occupational class upgrading with a polarised twist. Polarised upgrading was particularly strong in London, but also evident in Birmingham, Liverpool, Manchester or Newcastle. German cities experienced similar levels of occupational upgrading, but less polarisation. Notably in Berlin, Munich and Stuttgart, job growth was heavily concentrated among professionals and managers. While second-tier cities such as Dortmund, Essen or Leipzig also created many professional jobs, they experienced almost as much growth in low-end jobs. We find no evidence that job polarisation is specific to the largest cities or that strong job growth among professionals is limited to a few winner-take-all cities such as London and Berlin.

## Introduction

Since the early 1990 s, an influential argument in urban studies expected the labour markets of global cities such as Frankfurt, London or New York to become increasingly polarised, with job growth in high-wage professional and managerial occupations and in low-wage service occupations and a corresponding reduction in mid-level jobs (Sassen 1991). The polarisation thesis has prompted a host of empirical studies on the evolution of urban employment in cities such as London (Hamnett 2024; Morris 2025), Paris (Clerval 2022), Los Angeles (Scott 2019), New York and Tokyo (Van Ham et al. 2020). These studies show a common trend of growth in professional and managerial jobs across global cities, but much more variation in the expansion of low-end employment (Hamnett 2021).

The debate on urban polarisation in global cities echoes the controversy over occupational change at the national level (Fernández-Macías& Hurley 2017, Goos et al. 2009) and, more recently, at the regional level (Hurley et al. 2019; Morris and Oesch 2025) – with findings diverging between a trend towards occupational upgrading in Europe (Torrejon Pérez et al. 2025) and towards polarisation in the UK (Salvatori 2018) and the US (Autor 2019). Although these debates on urban and national labour markets were framed in similar terms, they have been conducted in isolation from each other.

Our study re-examines the polarised city thesis by extending the focus beyond a handful of global cities. We analyse change in the occupational class structure of the ten largest cities of Germany and the UK from 1991 to 2021 and systematically compare change at the city level to change at the country level. This allows us to test whether the occupational class structures of London and Berlin have evolved very differently to that of Leeds and Liverpool or Dortmund and Düsseldorf, and differently in large cities compared to the country as a whole.

Our study of ten major cities in both Germany and the UK therefore allows us to test the thesis of the uniqueness of global cities such as Berlin or London, defined as the command centres of the globalised economy that bring together the financial elite and low-paid migrants (Sassen 1991). It also permits us to see how well the thesis of the “great divergence” (Moretti 2012), which observes a growing gap in the economic fortune of American cities, translates from the United States to Western Europe. It may be just a few “winner-take-all” cities that have seen strong occupational upgrading, rather than large cities per se (Florida et al. 2020).

As the debate on polarisation is concerned with structural change, we look at a longer time frame than most studies, namely the three decades from the early 1990s to the early 2020s. Unlike analyses of ten years or less, this period is long enough not to be unduly influenced by the ups and downs of the business cycle. We use microdata from two large datasets: the censuses in 1991 and 2021 for England and Wales and a 2% random sample of workers registered in the social security system (SIAB) for Germany, 1993 and 2021. Both datasets allow us to produce results at the level of single cities.

Our study shows how the occupational class structure changed over time by distinguishing the upper-middle class of managers and professionals from the middle class of associate professionals and technicians, the lower-middle class of office clerks, the ‘old’ working class of production workers, and the ‘new’ working class of service and sales workers (Oesch 2006). Additionally, we use the analytical approach of job-quality quintiles (Wright and Dwyer 2003) as a robustness test, showing how employment changed over time across five hierarchically ordered quintiles based on median earnings of occupations. This makes it possible to define occupational change in terms of upgrading, downgrading and polarisation.

Our study provides new evidence on change in urban employment structures beyond global cities such as New York and London. It shows that the ten largest cities in Germany and the UK have all experienced strong growth in professional and managerial jobs. In both countries – and particularly in the UK – this growth in jobs at the top was accompanied by a rise in employment in low-paid service and sales jobs. However, this pattern of asymmetric polarisation, defined as strong job growth at the top, decline in the middle and moderate growth at the bottom, is not unique to the largest cities, but emerges as a broader post-industrial trend in the UK and, to a lesser degree, in Germany.

## Upgrading rather than polarisation of global cities

In the early 1990s, Sassen (1991) argued that the strategic hubs in the globalised economy, such as New York, Tokyo and London, but also Paris, Frankfurt, Hong Kong and São Paulo, were becoming increasingly polarised across occupational class lines. She expected the employment structure to expand among a transnational elite in financial and business services on the one hand, and low-wage migrants in personal services on the other, with a decline in middle-level jobs. Job growth at the two extremes of the occupational hierarchy was seen as interlinked, with high-wage professional jobs mechanically generating demand for low-wage personal service jobs (Sassen 1991). The mechanism is based on consumption spillovers: Professionals and managers in dual-earner households are time constrained but have purchasing power, buying time-intensive services from the market rather than producing them at home. Thus, rising demand for professionals and managers spills over into rising demand for personal service jobs in cleaning, eating out or childcare, which are often filled by migrants (Cerina et al. 2023; Estévez-Abe and Hobson 2015; Mazzolari and Ragusa 2013).

At the level of cities, the polarisation thesis has received mixed support (Hamnett 2021; Le Galès 2024). As case studies accumulated for cities such as Amsterdam (Boterman and Van Gent 2022), Hong Kong (Chiu and Lui 2004), Paris (Clerval 2022), Rotterdam (Custers and Engbersen 2022), Singapore (Baum 1999), Sydney (Baum 1997) and Toronto (Walks 2001), the principal finding is the disproportionate growth of professional and managerial jobs (Hamnett 2021). A comparative study of New York, Paris and Tokyo thus concludes that over the period 1980–2010 “professionalization of the workforce is the main trend of occupational structure change in all three cities” (Van Ham et al. 2020: 16). By contrast, polarisation, defined as the simultaneous growth of employment at both ends of the occupational structure, is “a contingent outcome in certain cities at certain times” (Hamnett 2021: 1050), such as London in the early 2000s.

London is a particularly interesting case. In the early 1980s, its labour market still had a larger working-class core than the rest of England. By the early 2000 s, however, it had a much larger proportion of (upper) middle class residents than other regions of the country (Butler et al. 2008; Hamnett 2003). Thereafter, the process of professionalisation slowed down, notably between 2001 and 2011 (Manley and Johnston 2014), before picking up again up to 2021. As a result, London experienced “asymmetric polarisation” (Hamnett 2024) in the first two decades of the 21st century, with strong employment growth among managers and professionals and weaker employment growth among routine workers and the casual self-employed.

While Hong Kong also showed a polarisation trend in the 1990s (Chiu and Lui 2004) and Stockholm in the 2000s (Nordin et al. 2025), most global cities seem to have experienced an upgrading of their employment structure (Van Ham et al. 2020). Table A.1 in Appendix A summarises the findings from twelve city case studies.

These case studies provide valuable evidence on how the class structure has changed in global cities. However, they cannot tell us whether the observed change is specific to global cities or whether it also applies to other large cities and countries more broadly. On this question, an influential argument from the United States expects very unequal fortunes across cities (Florida et al. 2020; Moretti 2012). Moretti (2012) showed that American cities with a more skilled workforce in 1980 greatly increased their advantage over cities with a less skilled workforce over the next three decades in terms of college graduates, earnings and even life expectancy. The more successful cities attracted not only more high-skilled workers in technology and finance, but also the “creative class” – knowledge workers and artists – who prefer to live in vibrant urban environments (Florida et al. 2020). As a result of this great urban divergence, it may be primarily a few “winner-take-all” cities that experience strong upgrading, defined as disproportionate growth in professional and managerial jobs, rather than large cities per se (Moretti 2012). Not only may larger American cities have seen more upgrading, they may also have experienced more polarisation. Several studies suggest that the growth in the employment share of high- and low-paid occupations relative to middle-paid occupations was stronger in larger US cities than in smaller ones (Autor 2019; Cerina et al. 2023; Parkhomenko 2025). Possible drivers include consumption spillovers – the increased demand from high-paid workers for low-skilled services (Cerina et al. 2023) – and rising house prices forcing middle-income workers to relocate to smaller cities in search of more affordable housing (Parkhomenko 2025). However, these findings of unequal urban fortunes were made for the American two-tier system of cities – and may not apply to Germany and the UK.

Indeed, an analysis of changes in the occupational class structure of Germany’s rural areas, small towns, large cities and metropolitan areas, based on the 1996–2018 microcensus, found a common shift towards an increasingly post-industrial employment structure, with the new salaried middle class and lower-skilled service occupations growing everywhere (Konietzka and Martynovych 2022). Another study for Germany, using fine-grained 1993–2019 social security data, found that “dominant cities” (cities with more than 600,000 inhabitants) experienced stronger occupational upgrading than both large and small cities, which in turn performed better than rural districts. For all geographical categories, outcomes were better in southern Germany than in north-western and especially eastern Germany (Westenberger 2025).

Empirical research on employment change at the country level in Western Europe also finds an upgrading trend in the occupational structure (Haslberger 2021; Oesch and Piccitto 2019; Torrejon Perez et al. 2025) and the class structure (Moawad and Oesch 2024), with a polarising twist in the UK (Goos and Manning 2007) that echoes findings for the United States (Autor 2019). These results suggest that countries, regions and cities may start out with a different occupational class structure. However, the evolution over time in the size of different occupational classes may be determined by similar long-term shifts in labour demand (due to technology and trade) and labour supply (due to educational expansion and increased female labour force participation). From this perspective, global cities and other large cities may not be unique in the evolution of their employment structure.

## City selection, data and analytical strategy

### City selection

Our analysis focuses on cities in Germany and the UK. While both countries have large market economies, their territorial organisation is very different. Strong political and financial federalism in Germany contrasts with strong centralism in the UK. This difference is also visible at the city level where the monocentric urban structure in the UK, dominated by London, contrasts with Germany’s polycentric urban structure, with important centres in the north (Hamburg), south (Munich), east (Berlin) and west (Frankfurt). The different role of the largest city is reflected in its contribution to national GDP: Greater London is responsible for 23 per cent, compared with 4 per cent for Berlin (OECD 2018: 21).

For both countries, we select the ten largest cities by population in 2021. In Germany, cities can be usefully defined by their municipal boundaries, making the selection of the ten largest cities straightforward: In both 1993 and 2021, they included Berlin, Hamburg, Munich, Cologne, Frankfurt, Stuttgart, Düsseldorf, Leipzig, Dortmund and Essen. Unlike German cities, British cities are under-bounded, with local authority boundaries often covering only parts of the actual city. For example, the contiguous economic area of Manchester extends well beyond the boundaries of Manchester City Council (Hamiduddin and Gallent 2012). For this reason, we use nine of the eleven cities that are part of the Core Cities group, a co-ordination mechanism set up in 1995 between the largest cities outside London. As our census data only covers England and Wales, we exclude two core cities: Belfast in Northern Ireland and Glasgow in Scotland. In addition to London, this leaves us with Birmingham, Bristol, Cardiff, Leeds, Liverpool, Manchester, Newcastle, Nottingham and Sheffield. We follow the city region descriptions of these ten cities and our city units in the UK thus reflect the metropolitan areas rather than the local authorities.^1^

Sassen (2007: 172) defines global cities as the command centres of the globalised economy that bring together the financial elite and low-paid migrants. While London appears to be the only global city in the UK, Sassen (1991, 2007) cites Berlin and Frankfurt as examples in Germany, although Hamburg and Munich could also be considered. Our analysis therefore focuses on the contrast between London and the other nine UK cities, while comparing the largest cities – Berlin, Hamburg, Munich, Frankfurt – with the rest in Germany.

### Data

In Germany, our analysis is based on individual-level data from the Integrated Labour Market Biographies (SIAB), 1993–2021 (Schmucker and Vom Berge 2023a, b). The SIAB provides a 2% random sample of all persons registered in the German social security system who are reported in the district in which they work, with large cities being districts. The SIAB sample includes employees, but excludes civil servants and the self-employed. While this exclusion is an obvious limitation, a constant share of the German labour force is excluded over the period that we study, with employees accounting for 83% of the labour force in 1993 and 82% in 2021.

We use the full sample provided by the SIAB which includes all individuals aged between 17 and 62 who are employed in a job subject to social insurance contributions. Following Dauth and Eppelsheimer (2020), we transform the daily spell structure of the SIAB into an annual sample and follow the convention of using 30th June as the cut-off date. This leaves us with analytical samples of *N* = 512,567 individuals in 1993 and *N* = 616,771 in 2021. We define occupations according to the 1988 German Classification of Occupations (KldB 88). After ensuring that the number of occupations is consistent over time, we are left with 114 occupations (see Appendix B).

For the UK, we use the 1991 and 2021 Censuses for England and Wales.^2^ The Censuses cover every individual present on 21 st April 1991 and 21 st March 2021 respectively, with individuals located in their place of residence. Individual-level data are not released until 100 years after collection, but area-level aggregates are available from Nomis (1991^3^) and the Office of National Statistics (2021^4^) respectively. As the 1991 Census offers a 10% sample and the 2021 Census a full sample, we multiply out the 1991 Census sample. Our analytical sample comprises all residents aged 16 or over who are employed or self-employed with known occupation. The 1991 and 2021 Censuses use different Standard Occupational Classification (SOC) schemes, respectively the 77 category SOC 1990 (SOC90) and the 104 category SOC 2020 schemes. We therefore use dual-coded Understanding Society data (University of Essex 2023), to harmonise SOC90 to SOC20 occupations (see Appendix B).

Our national data differ in two key characteristics: Geographically, they measure the place of work for Germany, but the place of residence for the UK. In terms of labour market coverage, they include the self-employed and civil servants in the UK, but exclude them in Germany. While it would be preferable to have fully harmonized data, this is not possible for the detailed geographical unit that our analysis requires, namely German districts and UK local authorities. However, note that our data are fully consistent within each country, providing us with reliable measures of occupational change over time.

### Analytical strategy

We analyse how the class structure of cities has changed by looking at absolute employment growth over the last three decades in five occupational classes. We follow an aggregated version of Oesch’s (2006: 132) class scheme, which distinguishes occupations vertically according to the skills required and horizontally according to the type of work logic (production logic, administrative logic or interpersonal service logic). This gives us the following five hierarchically ordered classes (see Appendix C for the coding of occupations into each class):

1. The upper and upper-middle class of managers and professionals;
2. The middle class of associate professionals, technicians and associate managers;
3. The lower middle class of administrative workers such as office clerks and secretaries;
4. The “old” working class of production workers: craft workers, plant operators, assemblers and agricultural workers;
5. The “new” working class of personal service and sales workers.

Table 1 shows the three largest occupations within each class in Germany and the UK in 2021 and the mean earnings by class, measured by hourly earnings in the UK and daily earnings in Germany. The mean earnings of each class are expressed relative to the national mean. The class differences in earnings are very similar in both countries. On average, professionals and managers earn the highest wages – at around 140% of the national mean – followed by associate professionals and technicians, whose earnings are close to the national mean. In both countries, clerical workers earn less than the national average but more than production workers, who in turn earn more than service and sales workers. In both countries, the latter earn only half as much as professionals and managers. These results suggest that our measure of occupational class captures a fundamental dimension of the labour market hierarchy, as reflected in earnings.

**Table 1:** Mean earnings and the three largest occupations in each class in Germany and the UK in 2021 (national mean earning = 100)

Class	DE		UK	
	Earning	Largest occupations	Earning	Largest Occupations
1. Upper-middle class: professionals and managers	143	IT professionals (Division) managers Engineers	140	Managers & directors Teachers IT professionals
2. Middle class: associate professional & technicians	102	Social workers Nurses Nursery teachers	103	Sales & marketing associate prof. Nurses Protective services
3. Lower middle class: office clerks	95	Office specialists Medical receptionists Office auxiliary workers	79	Other administrative occup. Secretaries Administrative occup. in finance
4. Old working class: production workers	85	Store & transport workers Elect. fitters & mechanics Warehousemen	76	Construction & building trades Agricultural & related trades Metal machining & fitting trades
5. New working class: service and sales workers	68	Sales assistants Motor vehicle drivers Cleaners	60	Caring personal services Sales assistants Road transport drivers

Data for earnings: DE SIAB 2021 (daily mean earnings); UK Annual Population Survey 2020-2022 (hourly mean earnings)

Data for largest occupations: DE SIAB 2021; UK England & Wales census 2021

## Results

### The class structure in major cities

**Fig. 1:**
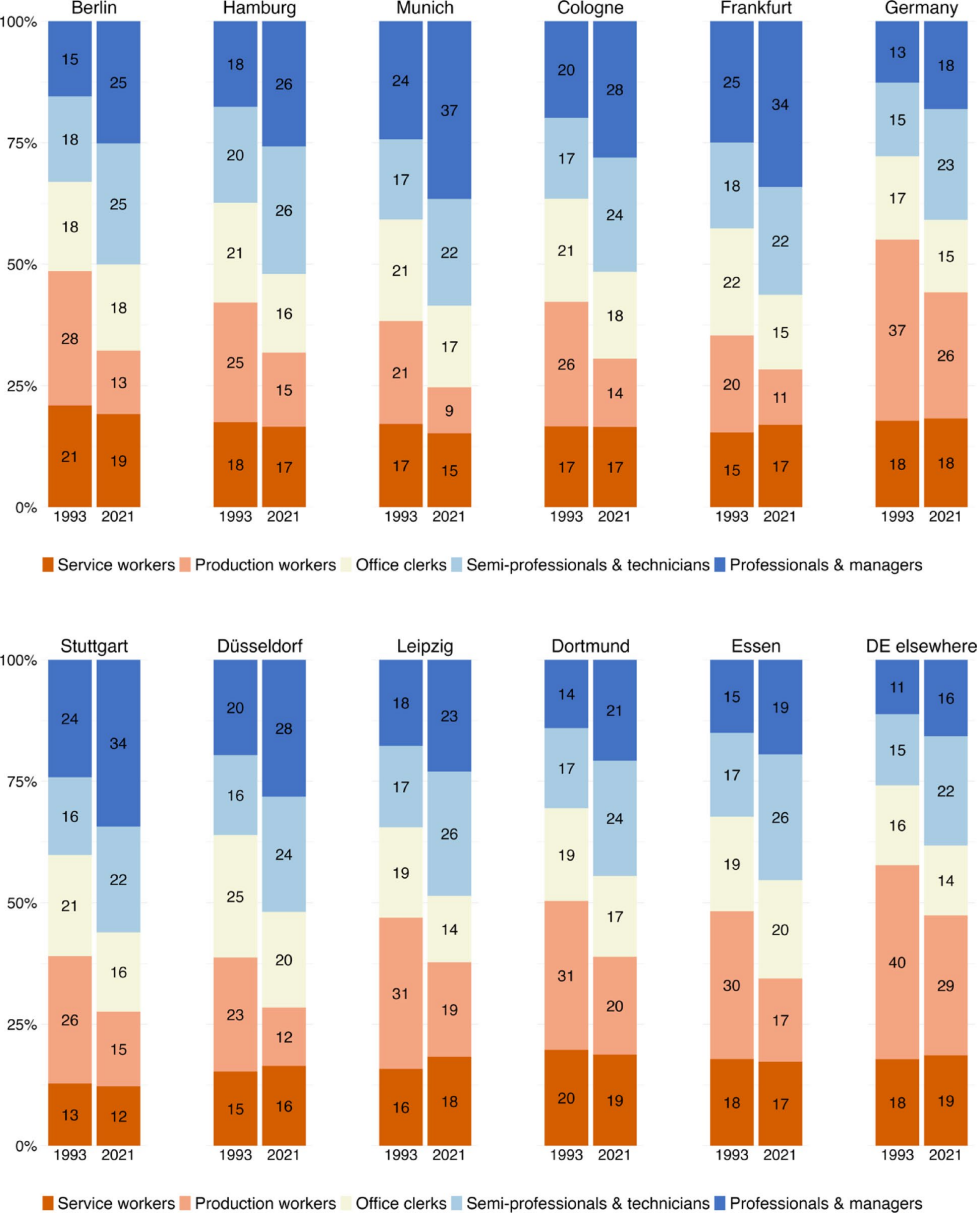
(**a**) the class composition in Germany in 1993 and 2021–1st to 5th largest cities. (**b**) the class composition in Germany in 1993 and 2021–6th to 10th largest cities

We begin our analysis by looking at the class structure of Germany’s ten largest cities. For the sake of comparison, we also show how the class structure changed in Germany as a whole (including the ten largest cities) as well as in the rest of Germany (excluding the ten largest cities and labelled “DE elsewhere”). Figure 1a and b show that, in both 1993 and 2021, large cities had much larger shares of professionals and managers – as well as associate professionals and technicians – than the country as a whole, and it was in these classes that the labour market expanded. Professionals, managers, associate professionals and technicians accounted for about a third of jobs in large cities in 1993. By 2021, their share had risen to 50 per cent. In 2021, the employment share of these occupational classes was particularly large in the affluent southern cities of Munich (59%), Frankfurt (56%) and Stuttgart (56%), but remained smaller in Dortmund (45%) and Essen (45%), two traditional industrial centres of the Ruhr area that retained large proportions of production workers. Consequently, production and service workers – the working class – accounted for over a third of employment in Dortmund (39%) and Essen (34%), but only a quarter in Munich (24%), Stuttgart (27%) and Frankfurt (28%).

Two special cases are the East German cities of Berlin and Leipzig, where the post-socialist transition was accompanied by a significant decline in the number of production workers. In Berlin in particular, the pace of occupational upgrading was remarkable. Whereas in 1993 Berlin was still dominated by working class jobs – with production and service workers accounting for half of employment (49%) – by 2021 the working class had shrunk to a third, and Berlin’s occupational class structure was no longer any different from that of Cologne, Düsseldorf or Hamburg.

**Fig. 2:**
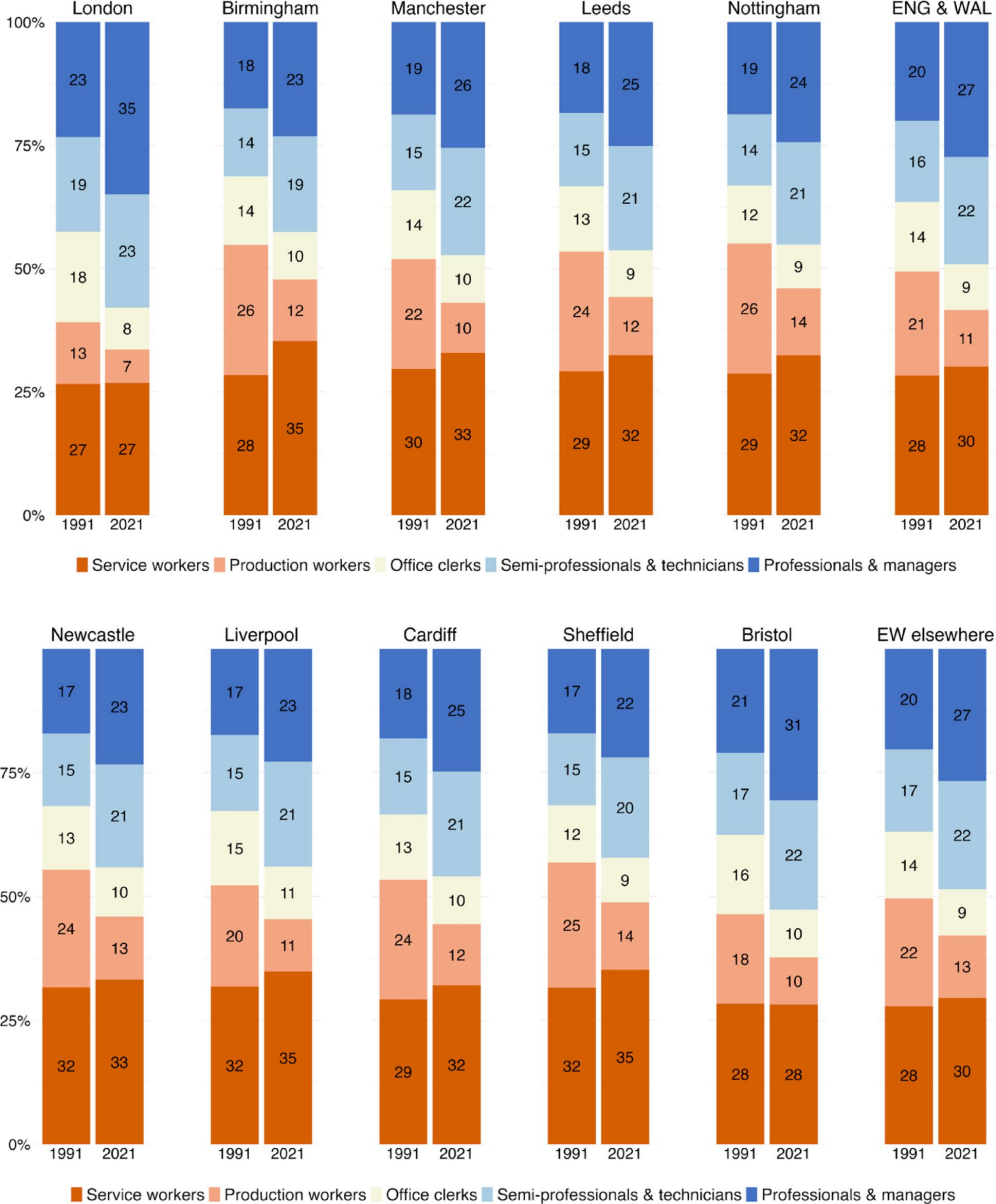
(**a**) the class composition in England & Wales in 1991 and 2021–1st to 5th largest cities. (**b**) the class composition in England & Wales in 1991 and 2021–6th to 10th largest cities

This similarity of German cities contrasts sharply with the situation in the UK, where Fig. 2a and b highlight London’s distinctive class structure. As in Germany, the UK also experienced class upgrading between 1991 and 2021. However, while professionals, managers, associate professionals and technicians increased their share of employment from 36 to 49 per cent in England and Wales, their share jumped from 42 to 68 per cent in London. The increase was particularly impressive at the top of the occupational hierarchy. By 2021, more than one in three economically active resident of London worked as professional or manager.

Outside London, the numerical importance of professionals and managers is much lower. Unlike Germany, England’s other large cities do not have a higher share of employment in these two occupations than the country as a whole (27%). The only major city with a higher proportion of professionals and managers in 2021 is Bristol (31%), while former industrial centres such as Birmingham, Liverpool, Newcastle or Sheffield have less than a quarter of employment in professional and managerial jobs, and thus less than the national average. Leeds and Manchester, while being slightly more middle class, resemble Sheffield much more than London. Unlike in Germany’s industrial cities, it is not production workers that account for the larger working class in England’s second-tier large cities, but service and sales workers who make up a full third of total employment. This is twice the size of the same class in Germany and demonstrates the post-industrial nature of the UK labour market - with a larger share of employment both among managers and professionals and among low-skilled service and sales workers.

### Employment growth and decline by class across cities

**Fig. 3:**
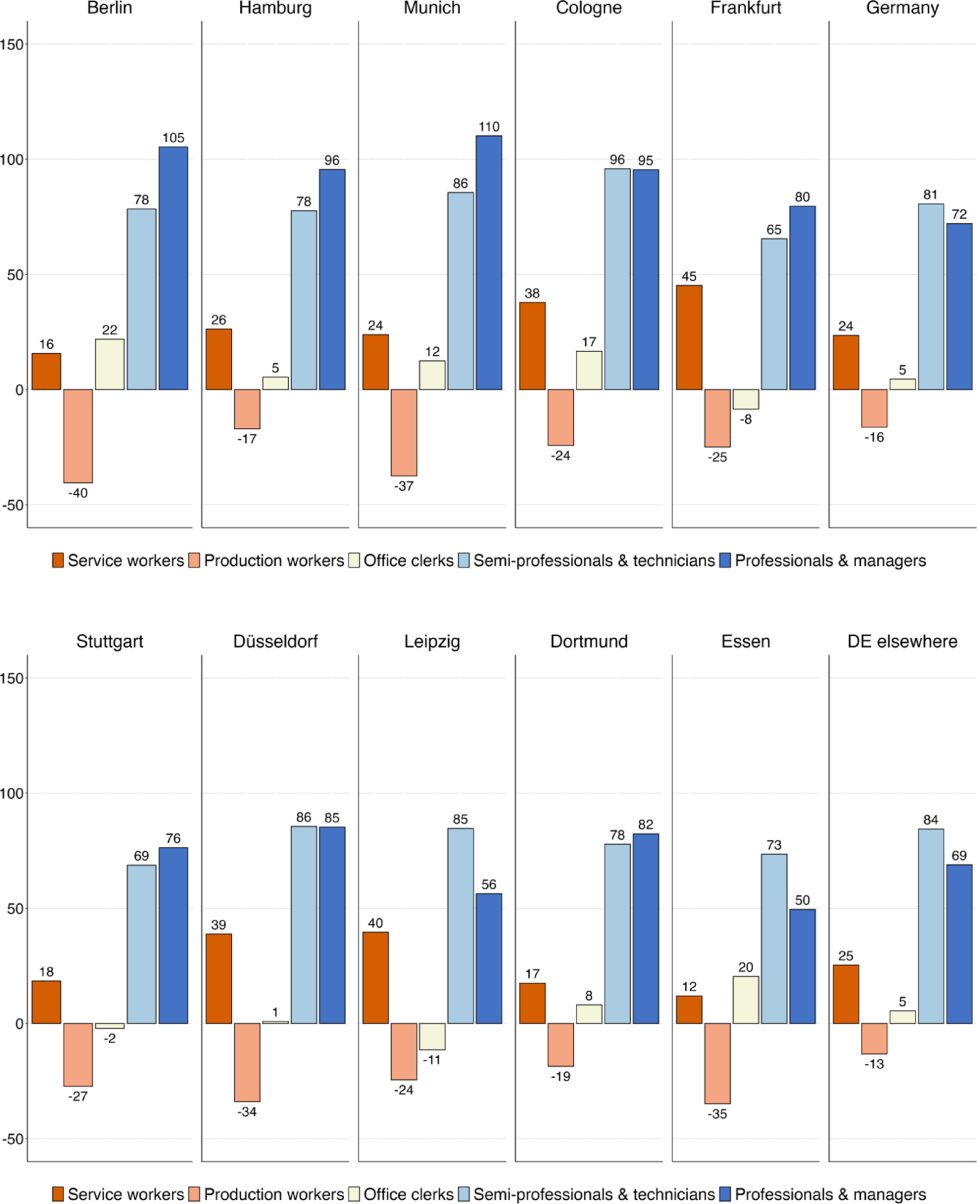
(**a**) Employment growth (in %) by class in Germany, 1993–2021–1st to 5th largest cities. (**b**) Employment growth (in %) by class in Germany, 1993–2021–6th to 10th largest cities

We shift the focus to the absolute change in employment across classes. Between 1993 and 2021, employment in Germany grew by 20 per cent, but this growth was very uneven across occupational classes (see Fig. 3a and b). In all the major cities, there was disproportionate growth among professionals and managers. Their employment expanded most strongly in the largest cities of Munich (110%), Berlin (105%), Hamburg (96%) and Cologne (95%) – and least in Leipzig (56%) and Essen (50%). By comparison, there is less variation in employment growth among associate professionals and technicians, with growth rates between 65 and 95 per cent. The employment of service and sales workers grew by between 15 and 25 per cent, the same rate as the German labour market as a whole – except in Cologne, Düsseldorf, Frankfurt and Leipzig, where the employment of service workers grew more strongly, leading to a slightly polarised pattern of occupational change. Across all cities, the employment of production workers declined substantially, more strongly so in the ten large cities than in the country as a whole (−16%), with the largest losses in Berlin (−40%) and Munich (−37%) and the smallest in Hamburg (−17%) and Dortmund (−19%).

**Fig. D.1:**
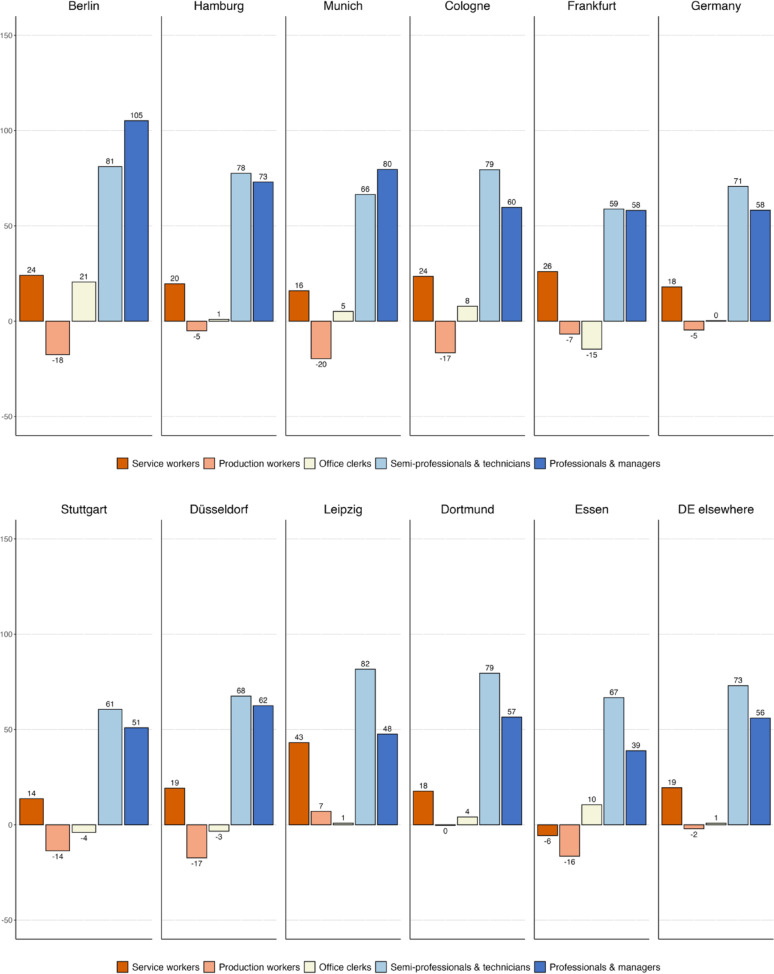
(**a**) Employment growth by class in Germany in %, 2000-2021–1^st^ to 5^th^ largest cities (**b**) Employment growth by class in Germany in %, 2000-2021–5^th^ to 10^th^ largest cities

We examine whether our findings for German cities and especially Berlin are unduly influenced by the post-reunification decade of the 1990s. To test this, we restrict our analysis to 2000–2021. The results are shown in Figures D.1a and D.1b in Appendix D and indicate that our conclusions are not driven by unusual shifts in the 1990 s and remain unchanged. Between 2000 and 2021, Germany’s ten largest cities and particularly Berlin have generated a disproportionate share of employment for professionals, managers, associate professionals and technicians, while jobs for production workers and office clerks have stagnated, with only small gains among service workers.

**Fig. 4:**
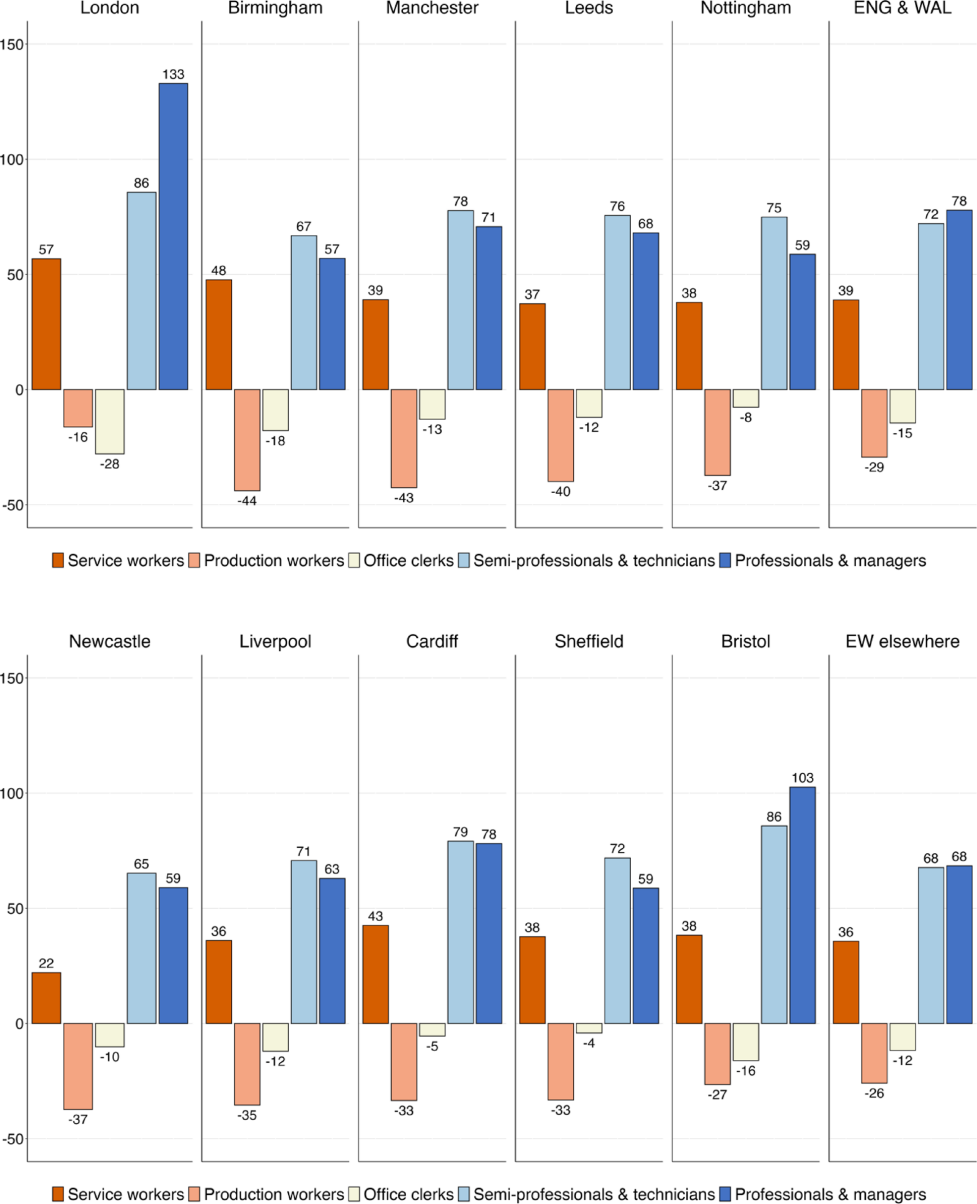
(**a**) Employment growth (in %) by class in England & Wales, 1991–2021–1st to 5th largest cities. (**b**) Employment growth (in %) by class in England & Wales, 1991–2021–6th to 10th largest cities

Figure 4a and b show employment change by class for England and Wales. Between 1991 and 2021, total employment increased by 31 per cent. All the ten largest cities in England and Wales experienced strongest job growth among professionals, managers, associate professionals and technicians. However, occupational upgrading was much stronger in London and, to a lesser extent, in Bristol than elsewhere. While employment in professional and managerial occupations more than doubled in London (133%) and Bristol (103%), job growth in the same occupations was only half as strong in Birmingham (57%), Newcastle (59%) and Sheffield (59%). By and large, the pattern of class change looks very similar in the largest cities outside London and Bristol as in the rest of England and Wales.

In the UK, not only the (upper-)middle classes experienced job growth, but also lower skilled workers in service and sales jobs. Differences in growth rates across cities are much smaller for service workers: Birmingham (48%) saw a similar employment increase in this class as London (57%), with the other cities experiencing growth rates of around 35 to 40 per cent (Newcastle being an outlier with only 22%). As a result, we observe asymmetrical polarisation in all of England’s ten largest cities, with strong job growth at the top, moderate growth at the bottom and declining employment in the middle of the class hierarchy.

Looking at the declining classes, one of the most striking differences with Germany is the shrinking employment of office clerks in England, which fell by 15 per cent between 1991 and 2021, compared with a modest increase of 5 per cent in Germany. While this decline can be seen across large cities in England and Wales, it is particularly pronounced in London, where the number of clerical workers decreased by almost 30 per cent. In the other nine cities, the class with the largest employment decline was not office clerks, but production workers. In former industrial centres such as Birmingham (−44%), Leeds (−40%) and Manchester (−43 per cent), this decline was larger than for the country as a whole (−29 per cent). As in Germany, large cities in England and Wales have de-industrialised faster than the rest of the country.

**Fig. D.2:**
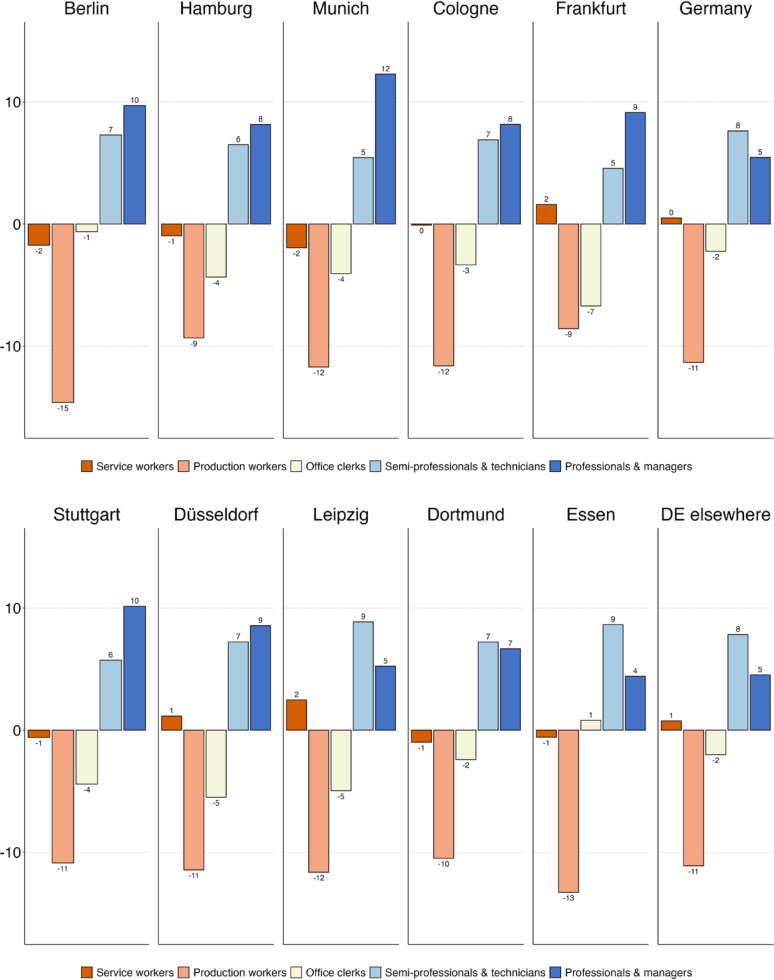
(**a**) Employment change by class in Germany in pp, 1993-2021–1^st^ to 5^th^ largest cities (**b**) Employment change by class in Germany in pp, 1993-2021–6^th^ to 10^th^ largest cities

So far, our analysis has shown the employment growth of each occupational class in per cent. Although these classes were similar in size in the early 1990 s, there was some variation between the smallest (office clerks) and largest class (production workers in Germany, service workers in the UK). To account for these different baselines, we show change in percentage points in Figures D.2a–b and D.3a–b (Appendix D). To better illustrate the shifts in distribution, these Figures show the change in percentage points after factoring out the overall employment growth over the period (20% in Germany, 31% in the UK), the percentage point shifts across classes thus adding up to zero.

These results point to very similar conclusions. In Germany, production workers saw the greatest employment declines across all cities (–9 to − 15 percentage points). By contrast, professionals and managers recorded the greatest growth in employment share (+ 8 to + 12 percentage points) in the seven largest cities, while associate managers and technicians contributed more to job growth in the 8th to 10th cities—Leipzig, Dortmund, and Essen. Interestingly, the employment share of service workers stagnated, falling by 2 percentage points in Berlin and Munich, and rising by two points in Frankfurt and Leipzig. This challenges the view that over the last few decades Germany’s labour market has become polarised due to strong consumption spillovers.

For England and Wales, these Figures show that professionals and managers contributed more to employment growth than associate professionals and technicians only in London and Bristol. In the other eight cities, both groups saw similar increases in their employment share of five to seven percentage points. It is noteworthy that once overall employment growth is factored out, the employment share of service workers did not increase in London or Bristol and rose only slightly across England and Wales. However, in poorer cities such as Birmingham, Nottingham, and Sheffield, the employment share of this low-paid occupational class grew by four to six percentage points, producing a more polarised pattern of occupational change.

### Employment growth in quintiles across cities

The interpretation of employment change in terms of upgrading, downgrading and polarisation assumes a linear hierarchy of more or less rewarding occupations. However, while our five classes are broadly hierarchically ordered, they may conceal considerable heterogeneity in occupations. Production workers can be employed in well-paid occupations such as machine mechanics or in low-paid occupations such as agricultural labourers – as can service workers employed as flight attendants or domestic helpers. For this reason, we replicate our class-based analysis using the quintile approach (Wrigth and Dwyer 2003).

This approach consists of ranking all occupations by their median wages and then dividing these rank-ordered occupations into five groups containing each one fifth of total national employment at the earliest point in time: 1991 for England and Wales and 1993 for Germany. We construct these quintiles on the basis of national employment (using the same analytical sample as above) so that occupations within each country always fall into the same quintile, allowing meaningful comparisons between cities. The bottom and top national quintiles therefore include the 20% of workers in the lowest and highest paid occupations respectively. This allows us to follow the evolution of employment across these five hierarchically ordered quintiles in each city and to express occupational change in terms of upgrading (growth in the top quintile), downgrading (growth in the bottom quintile) or polarisation (growth at both ends).

**Fig. 5:**
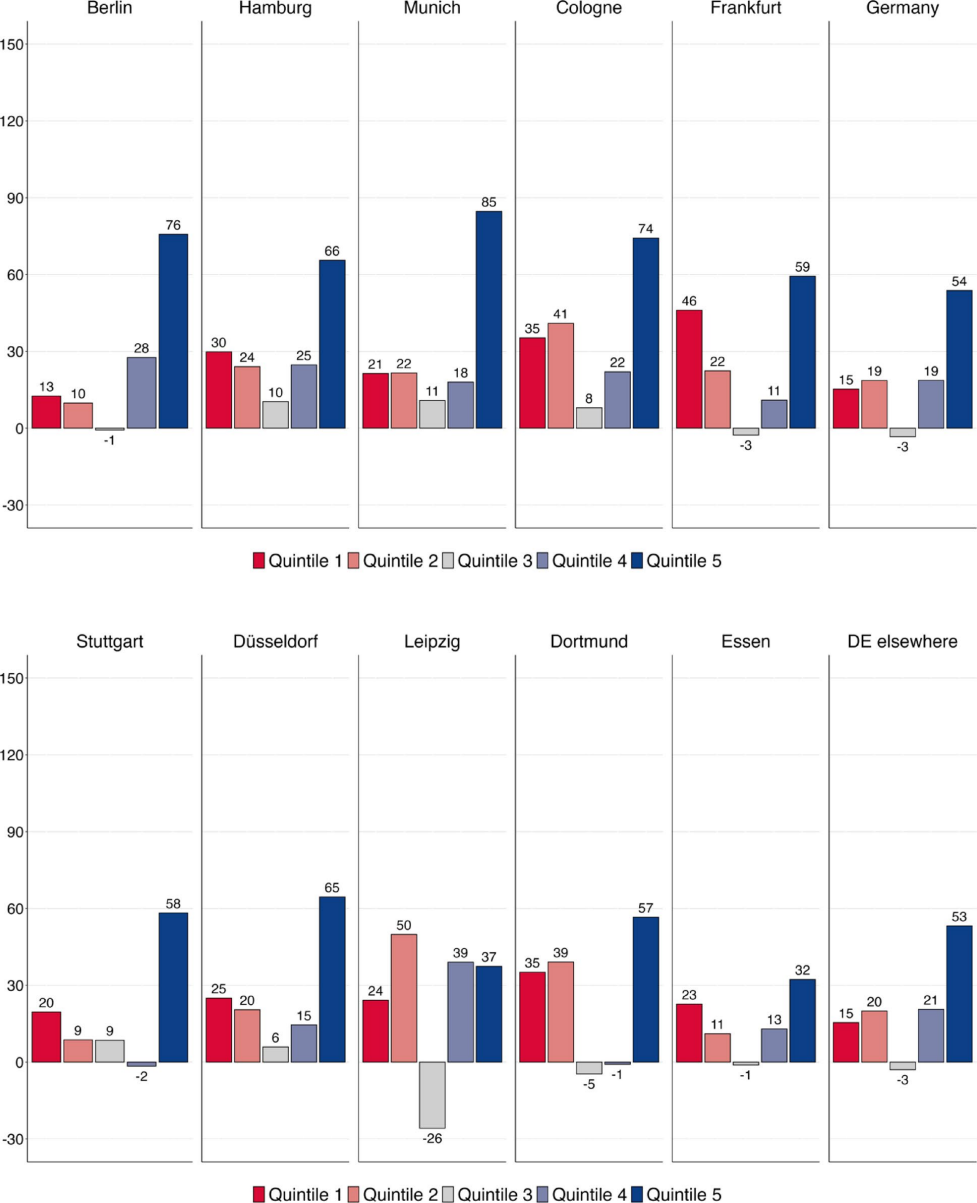
(**a**) Employment growth (in %) by quintile in Germany, 1993–2021–1st to 5th largest cities. (**b**) Employment growth (in %) by quintile in Germany, 1993–2021–6th to 10th largest cities

**Fig. D.4:**
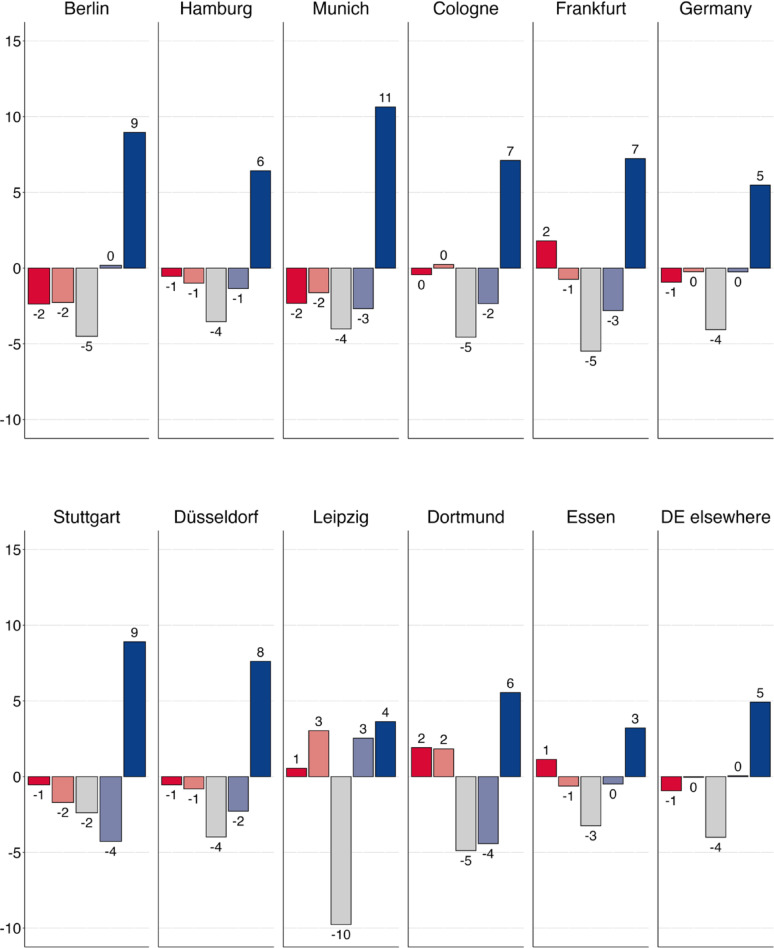
(**a**) Employment change by quintile in Germany, 1993-2021–1^st^ to 5^th^ largest cities (**b**) Employment change by quintile in Germany, 1993-2021–6^tht^ to 10^th^ largest cities

Figure 5a and b replicate the class-based analysis and document employment growth across the five quintiles for Germany’s ten largest cities between 1993 and 2021 (these results show absolute employment growth in per cent – for relative employment growth in percentage points, see Figures D.4a–b and D.5a–b (Appendix D). Again, we find occupational upgrading to be the dominant trend in Germany, with employment growth by far the strongest in the highest paid occupations set in quintile 5 (Q5). However, given that employment stagnated in the middle quintile Q3, while it expanded in the two bottom quintiles Q1 and Q2, there is a polarising twist to the upgrading of the employment structure. This applies particularly to Dortmund, Frankfurt and Leipzig. By contrast, in Hamburg, Stuttgart and, most notably, in Munich and Berlin, massive job growth in the top quintile dwarfed weak job growth in the two bottom quintiles. In this sense, the largest cities of Germany were least likely to have experienced a polarization of their employment structure over the last three decades.

**Fig. 6:**
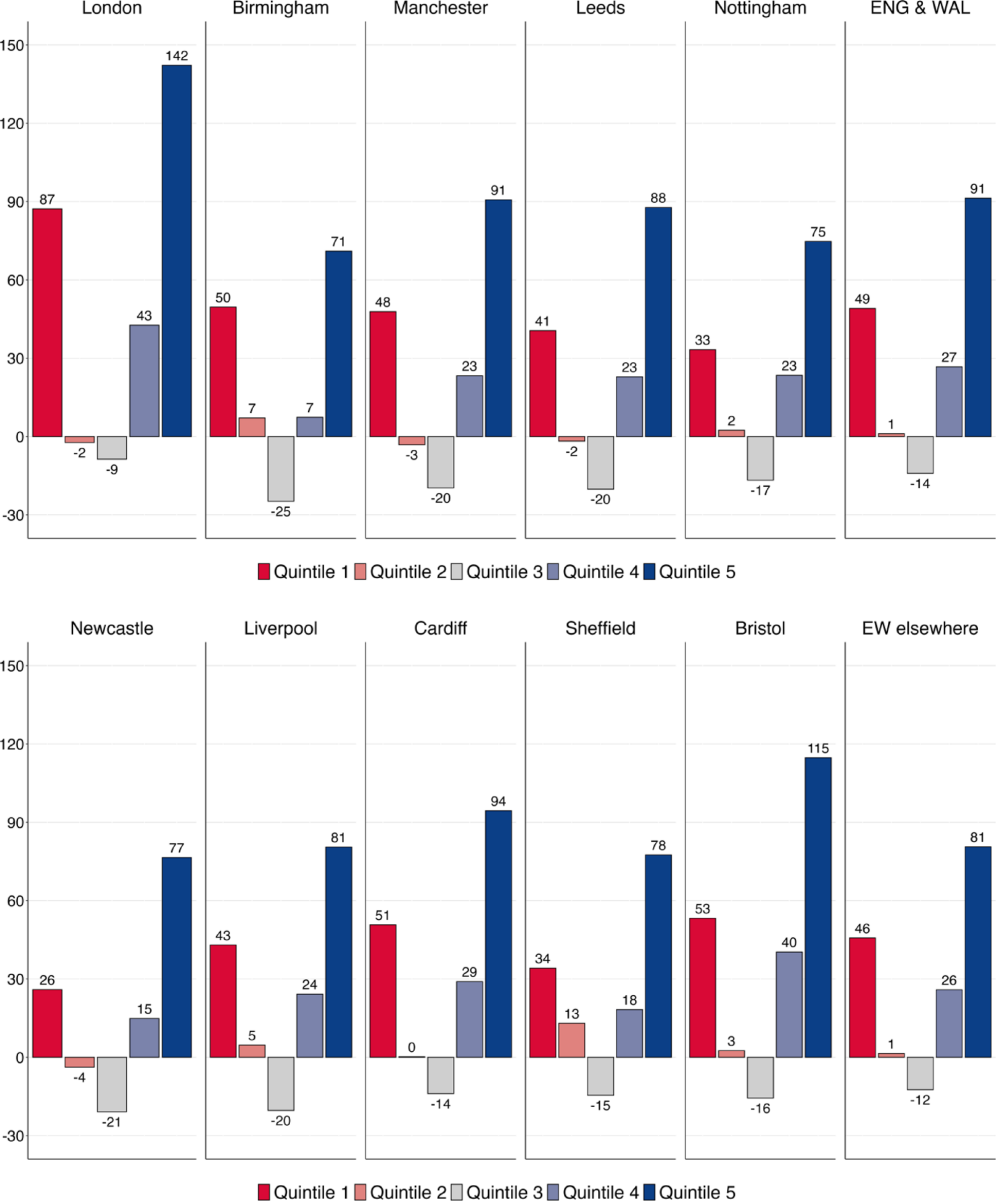
(**a**) Employment growth (in %) by quintile, England & Wales 1991–2021–1st to 5th largest cities. (**b**) Employment growth (in %) by quintile, England & Wales 1991–2021–6th to 10th largest cities

Figure 6a and b extend the analysis by quintiles to England and Wales. For once, it is not the differences that are striking, but the similarities between cities. In every single city, employment growth was strongest in the top quintile 5, followed by the bottom quintile 1, second-to-top quintile 4 and second-to-bottom quintile 2. While these four quintiles all experienced employment growth over the past three decades, there has been an erosion of employment in the middle quintile 3, most strongly in Birmingham. As a result, in all major cities in England and Wales we observe asymmetric polarisation, with stronger job growth at the top than at the bottom and a decline in jobs in middle occupations. Although absolute employment growth was much stronger in London than in Liverpool, Newcastle, Nottingham or Sheffield, the relative pattern of job change was the same.

## Discussion and conclusion

This article has revisited the polarised city thesis by analysing change in the occupational class structure of the ten largest cities in Germany and the UK between 1991 and 2021. Based on census and administrative data, it has produced four main findings.

First, our analysis provides partial support for the polarised city thesis in the UK. London, the archetypal global city, has experienced strong upgrading – with growth in professional and managerial jobs – alongside moderate expansion of lower-skilled service and sales work and a sharp decline in clerical employment. Although jobs have grown at both ends of the class hierarchy, growth at the top has far outpaced that at the bottom. The result is polarised upgrading, or what Hamnett (2024) called the “asymmetric polarisation” of London’s class structure. Change in the occupational structure looks similar in other major cities in England and Wales: strong growth among professionals, managers, associate professionals and technicians; moderate growth among service and sales workers; and decline among production workers and office clerks. Consequently, cities such as Birmingham, Leeds, Liverpool, Manchester and Newcastle have also been upgrading with a polarising twist. What sets London apart is the exceptionally strong expansion of professional and managerial jobs, with annual growth exceeding four per cent.

Second, we find weaker evidence of polarisation in Germany, where occupational upgrading has been consistent across cities, with Berlin and Munich showing the strongest growth in professional and managerial jobs. German cities created fewer low-skilled service jobs than large UK cities and lost fewer clerical jobs. Although many mid-paid production jobs disappeared, the upgrading trend is stronger and the polarisation trend weaker in Germany than in the UK. Some differences remain: while Berlin, Munich and Stuttgart added many jobs at the top of the class hierarchy and few at the bottom, Dortmund, Frankfurt and Leipzig saw notable growth at the bottom. Consequently, the labour markets of the three largest German cities have polarised less than those of other large cities in Germany. A similar observation applies to the UK. Unlike Birmingham and Leipzig, growth in managerial and professional jobs far outpaced growth in sales and service work in London, Berlin and Munich, tilting occupational change toward upgrading rather than polarisation. These findings contrast with studies of the United States that suggest larger cities experienced stronger polarisation than smaller ones (Autor 2019; Cerina et al. 2023; Parkhomenko 2025). Our analysis, however, is limited to small samples of the ten largest cities in each country.

Third, our results refute the “winner-take-all” thesis for large cities in both Germany and the UK. Contrary to Moretti’s (2013) findings for the US, poorer cities in the 1990 s, such as Leipzig in East Germany or Dortmund in the Ruhr area, were not left behind in terms of occupational upgrading. Although Dortmund and Leipzig still had a larger working-class base in 2021, they did not experience a much lower rate of job growth among professionals, managers and technicians than richer cities such as Frankfurt or Stuttgart. Likewise, the similarity in occupational change between Birmingham and Nottingham, Cardiff and Bristol, Manchester and Sheffield contradicts the narrative of great urban divergence for the UK.

Fourth, our analysis also contributes to the debate between occupational upgrading and polarisation at the national level. Our data provides some evidence for both perspectives. In all cities and both countries, the upper-middle class of professionals and managers experienced the strongest employment growth, followed by the middle class of associate professionals and technicians. This result supports the case for upgrading. At the same time, deindustrialisation and the computer revolution have made middle-skilled – or at least middle-paid – production jobs (in German cities) and back-office jobs (in British cities) harder to find. Meanwhile, with sustained growth in low-paid service and sales employment, notably in the UK, upgrading has become polarised. However, polarised upgrading is not a specific feature of large cities, but applies more generally to post-industrial UK and, to a lesser extent, Germany.

## Figures and Tables

**Fig. D.3:**
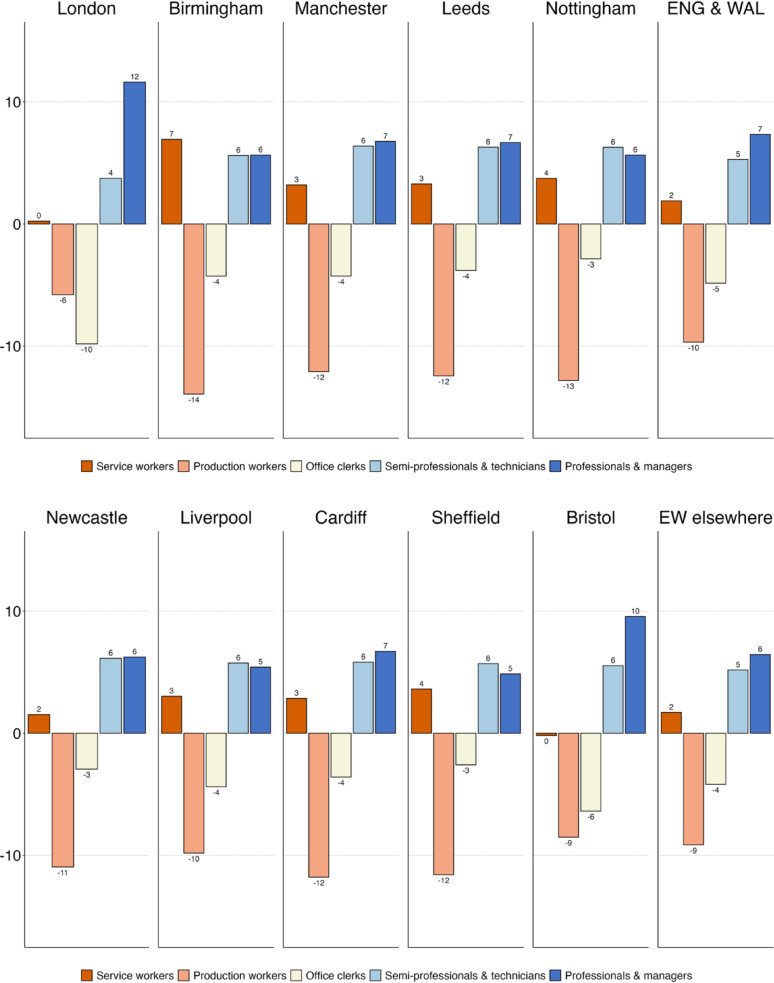
(**a**) Employment change by class in England & Wales in pp, 1991-2021 - 1^st^ to 5^th^ largest cities (**b**) Employment change by class in England & Wales in pp, 1991-2021–6^th^ to 5^th^ largest cities

**Fig. D.5:**
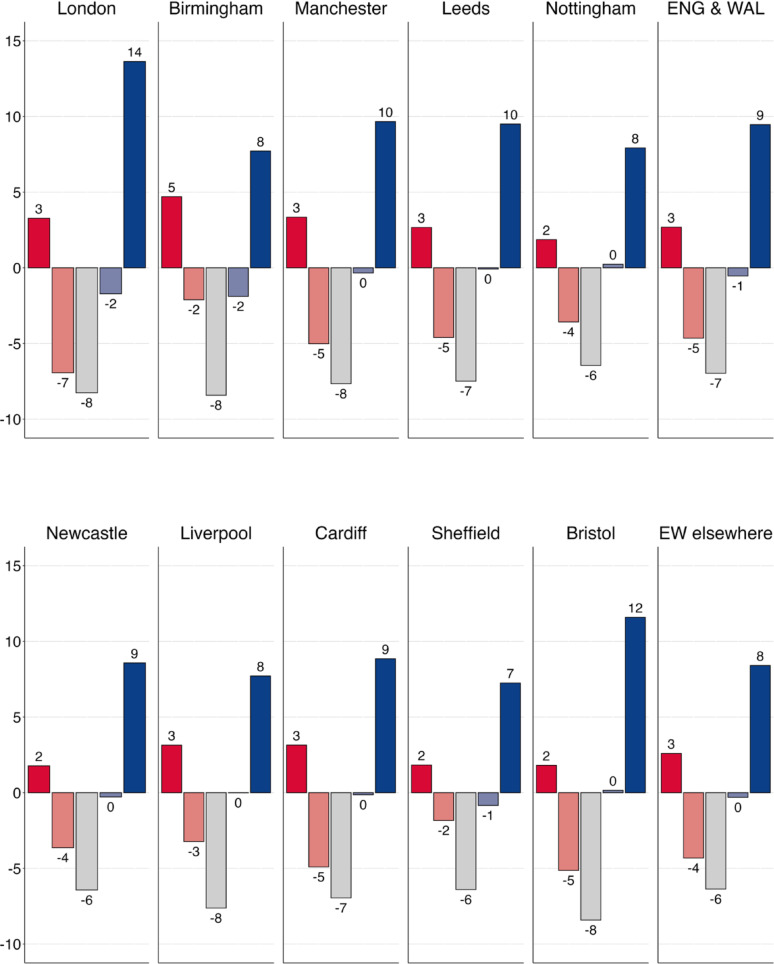
(**a**) Employment change by quintile in England & Wales, 1991-2021 - 1^st^ to 5^th^ largest cities (**b**) Employment change by quintile in England & Wales, 1991-2021–5^th^ to 10^th^ largest cities

## Data Availability

This study is based on two public data sources: German SIAB micro data are based on social security records and available at: https://fdz.iab.de/en/our-data-products/individual-and-household-data/siab/eurostat/web/microdata/european-union-labour-force-survey- Local census data for England and Wales are available at: Census 1991: https://www.nomisweb.co.uk/query/construct/summary.asp?mode=construct&version=0&dataset=35. Census 2021: https://www.ons.gov.uk/datasets/create.

## Appendix A

See (Table A.1).

**Table A.1:** Empirical studies of occupational change in global cities, 1999–2024

Author(s)	City	Period covered	Data source	Finding
Baum (1999)	Singapore	1983–1996	Singapore Department of statistics	Professionalization without polarisation, with a growing middle class.
Butler et al. (2008)	London	1981–2001	UK Census (1981, 1991, 2001)	professionalization and class upgrading, with decrease among lower professional and intermediate non-manual groups.
Chiu & Lui (2004)	Hong Kong	1991–2001	Population census (1991, 1996, 2001)	Polarised professionalisation: growth among managers and professionals. Decline among production workers. Moderate increase among service workers
Clerval (2022)	Paris	1982–2008	French Census (1982, 1990, 1999, 2008)	Growth in professional and managerial class, decline in working-class population
Custers and Engbersen (2022)	Rotterdam	2008–2017	Rotterdam Neighborhood Profile survey (2008, 2017)	Growth in middle classes with high cultural capital he expense of the lower and middle classes with low cultural capital
Hamnett (2015)	London	1961–2011	UK Census (2001, 2011)	Long-term shift towards professionalization rather than polarisation, with middle-class expansion slowing down in the 2000s
Hamnett (2024)	London	2001–2021	UK Census (2001, 2011, 2021)	Continued professionalization but also asymmetric polarisation, with self-employed and routine workers increasing.
Manley and Johnston (2014)	London	2001–2011	UK Census (2001, 2011)	No strong evidence of working-class decline in London, but stability in working-class share
Nordin et al. (2025)	Stockholm	2007–2019	Register data on private firms	Polarization with private-sector jobs growing in high-skill and low-skill industries
Scott (2019)	Los Angeles	2000–2015	County Business Patterns, US Census	Gentrification driven job growth in knowledge-intensive white-collar jobs
van Ham et al. (2020)	New York, London, Tokyo	1980–2010	National census data from US, UK, Japan	Professionalization dominates in all three cities, with high-income job growth and middle-income stability
Walks (2001)	Toronto	1971–1991	Canadian Census (1971, 1991)	Strong professionalization and decline in manufacturing jobs, moderate increase in low-level services jobs

## Appendix B

### Germany: harmonising the KldB 1988 occupations

In order to ensure a time-consistent number of occupational groups, six occupational groups that were only declared in 1993 but not in 2021 were merged with another related occupational group. We have collapsed “Drillers up to borers” (15) with “Toolmakers up to precious metal smiths” (28), “Locksmiths, not specified up to sheet metal, plastics fitters” (22) with “Plant fitters, maintenance fitters up to steel structure fitters, metal shipbuilders” (24), “Metal workers (no further specification)” (35) with “metal polishers up to metal bonders and other metal connectors” (17), “Packagers, good receivers, despatchers” (55) with “Stowers, furniture packers up to stores/transport workers” (86), “Assistants (no further specification)” (56) with “Building labourer, general up to other building labourers, building assistants” (47), and “Cashiers” (91) with “Salespersons” (73).

### UK: harmonising to SOC20 occupations

We harmonise SOC90 to SOC20 occupations by identifying individuals present in Understanding Society survey waves where occupations were coded to more than one SOC classification scheme, for example SOC90 and SOC00. For all employed respondents with dual-coded SOC90 and SOC00 data, we first identify the proportions in which each SOC90 code splits into SOC00 codes. We use these splits to recode Census 1991 SOC90 occupations to SOC00 occupations, assuming that Understanding Society data are representative of broader reallocations. We then do the same to harmonise SOC00 to SOC10 occupations, and SOC10 to SOC20 occupations.

## Appendix C

See (Table C.1).

**Table C.1:** Allocation of occupational codes into classes in Germany and the UK

Class	Occupation codes Germany (SIAB – KldB 1988)	Occupation codes UK (Censuses, SOC20)
1. Service workers	40, 73, 74, 81, 96, 105, 114, 115, 116, 117, 118, 119, 97, 120	543, 611, 612, 613, 621, 622, 623, 624, 625, 711, 712, 721, 821, 822, 823, 921, 922, 923, 924, 925, 926
2. Production workers	1, 2, 3, 4, 5, 7, 8, 9, 10, 11, 12, 13, 14, 16, 17, 18, 19, 20, 21, 23, 24, 25, 26, 27, 28, 30, 31, 32, 33, 34, 36, 37, 38, 39, 41, 42, 43, 44, 45, 46, 47, 48, 49, 50, 51, 52, 53, 54, 57, 58, 85, 86, 6, 80, 84	511, 521, 522, 523, 531, 532, 541, 542, 544, 811, 812, 813, 814, 815, 911, 912, 913
3. Office clerks	83, 93, 94, 95, 107	411, 412, 413, 414, 415, 421
4. Associate professionals & technicians	79, 29, 64, 66, 67, 68, 70, 71, 72, 75, 78, 82, 90, 98, 100, 101, 103, 104, 106, 108, 109, 110, 112, 69	115, 121, 122, 222, 223, 247, 312, 313, 321, 322, 323, 324, 331, 341, 342, 343, 351, 352, 353, 354, 355, 356, 357, 358, 524, 525, 533, 631, 713, 722, 816
5. Professionals & managers	59, 60, 61, 62, 63, 65, 76, 77, 87, 88, 89, 92, 99, 102, 111, 113, 114	111, 112, 113, 116, 117, 123, 124, 125, 211, 212, 213, 214, 215, 216, 221, 224, 225, 231, 232, 241, 242, 243, 244, 245, 246, 248, 249, 311

## Appendix D

### Additional analyses

- Figure D.1a: Employment growth by class in Germany in %, *2000*–2021–1st to 5th largest cities.
- Figure D.1b: Employment growth by class in Germany in %, *2000*–2021–5th to 10th largest cities.
- Figure D.2a: *Relative employment change* by class in Germany *in pp*, 1993–2021–1st to 5th largest cities.
- Figure D.2b: *Relative employment change* by class in Germany *in pp*, 1993–2021–6th to 10th largest cities.
- Figure D.3a: *Relative employment change* by class in England & Wales *in pp*, 1991–2021–1st to 5th largest cities.
- Figure D.3b: *Relative employment change* by class in England & Wales *in pp*, 1991–2021–6th to 5th largest cities.
- Figure D.4a: *Relative employment change* by quintile in Germany *in pp*, 1993–2021–1st to 5th largest cities.
- Figure D.4b: *Relative employment change* by quintile in Germany *in pp*, 1993–2021–6^tht^ to 10th largest cities.
- Figure D.5a: *Relative employment change* by quintile in England & Wales *in pp*, 1991–2021–1st to 5th largest cities.
- Figure D.5b: Employment change by quintile in England & Wales, 1991–2021–5th to 10th largest cities.

#### Figures D.1: employment change in Germany over a shorter period (2000–2021)

See Figs. D.1, D.2, D.3, D.4 and D.5.

## Footnotes

1 For example, London comprises the 32 London boroughs and the City of London. Likewise, Manchester comprises Manchester City Council and the 9 metropolitan boroughs that form the Greater Manchester Combined Authority.

2 The Scottish Census was delayed until March 2022 due to COVID-19. To date, 2022 Scottish Census results have only been released for grouped (10 category or 27 category) occupations, which is an insufficient level of detail to construct occupational class groups.

3 NOMIS 1991 census - local base statistics; Table L99 Occupation; Standard Occupational Classification (10% sample): Residents aged 16 and over, employees and self-employed; available from https://www.nomisweb.co.uk/query/construct/summary.asp? mode=construct&version=0&dataset=35.

4 Office for National Statistics (ONS) 2021: Census 2021; All usual residents; Occupation (current) 105 categories; available from https://www.ons.gov.uk/datasets/create.
